# House-Building Materials

**Published:** 1875-01

**Authors:** 


House Building Materials.—Bricks in Pharaoh's day were hardened in the sun, which might answer very well in a country where there was no rain and where there were no houses higher than one story. The inventor of the first brick kiln may never be known, but the hardening process of fire makes brick the best material for building purposes yet devised. Brick resists fire better than iron or solid granite, and have more strength to bear weight than many kinds of stone. A good American brick will bear a weight of fifty thousand pounds before it cracks, or is crushed. Some English bricks have borne a pressure of a quarter of a million pounds without injury.

Iron is supposed to be very brittle in winter; it will suspend a weight as heavy in winter as in summer; it is only when it is jarred, by being struck with a hammer, or thrown down on any hard substance, that it is easily broken when intensely cold. It is broken only through shock, and not when exposed to steady strain. To persons building houses in which they expect to live the remainder of their days, a knowledge of the strength of brick and iron is important.

If a brick house is burned down the loss is less than if built of iron or stone, because the stone chips off or crumbles, and the iron becomes so bent as to be entirely useless; brick can be built into the wall again, because just as good as they were before the fire.

